# The Influence of Nursing Leadership Styles on Medication Safety: A Mixed‐Methods Systematic Review

**DOI:** 10.1111/inr.70166

**Published:** 2026-02-23

**Authors:** Hasan Fehmi Dirik, Chrysanthi Theiakouli, Nikolaos Efstathiou

**Affiliations:** ^1^ Faculty of Nursing Dokuz Eylul University Izmir Turkey; ^2^ Department of Nursing National and Kapodistrian University of Athens Athens Greece; ^3^ Department of Nursing and Midwifery University of Birmingham Edgbaston Birmingham UK

**Keywords:** Leadership, medication errors, nursing, systematic review

## Abstract

**Aim:**

To synthesise evidence on the impact of nursing leadership styles on medication errors.

**Background:**

Leadership plays a critical role in medication safety by shaping staff behaviour, promoting adherence to clinical guidelines and fostering a safety‐oriented culture. However, the effects of specific leadership styles on medication safety have not yet been comprehensively synthesised.

**Methods:**

The Joanna Briggs Institute convergent segregated approach for mixed‐method systematic reviews was employed and reported in accordance with PRISMA guidelines. Searches were conducted in MEDLINE, CINAHL, Scopus, PubMed, Web of Science, EMBASE, PsycINFO and Google Scholar, encompassing literature published from January 2014 to April 2025.

**Results:**

The review includes 15 papers: 11 cross‐sectional, 2 quality improvement, 1 quasi‐experimental and 1 mixed‐methods study. Transformational (*n* = 11) and ethical leadership styles were associated with enhanced medication safety, as evidenced by increased error reporting and greater adherence to medication protocols. Situational leadership was linked to gradual improvements in self‐reporting behaviours. In contrast, passive‐avoidant and toxic leadership styles correlated with higher rates of medication errors and decreased willingness to report errors. The effects of transactional leadership (*n* = 5) were mixed and appeared to be influenced by contextual factors.

**Discussion:**

Synthesising both consistent and divergent findings clarified the complex dynamics through which leadership can mitigate or exacerbate medication errors, and offered guidance for the development of innovative and integrated leadership approaches.

**Conclusion:**

Transformational, ethical and situational leadership styles contribute to improved medication safety, whereas passive‐avoidant and toxic leadership styles lead to increased medication errors.

**Implications for Nursing and Nursing Policy:**

This review highlights the relevance of leadership approaches associated with safety‐oriented behaviours. Understanding the effects of different leadership styles will inform and support decision‐making, enabling nurse leaders to implement more effective strategies to reduce medication‐related risks. Investment in leadership development, particularly programmes based on transformational and ethical principles, has the potential to strengthen safe medication practices.

**PROSPERO Registration:**

This systematic review's protocol was registered in PROSPERO (registration number CRD42023380426).

## 1 Introduction

The World Health Organisation (WHO 2019) estimates that one in ten patients in high‐income countries is harmed during hospital care. In low‐ and middle‐income countries (LMICs), approximately 134 million adverse events occur annually, resulting in 2.6 million deaths. Medication errors are major contributors to this harm. Globally, one in twenty patients experiences preventable medication‐related harm, with over one‐quarter of these cases classified as severe or potentially life‐threatening and having a high financial cost (WHO 2017, 2024). These errors occur at various stages of the medication process – most commonly during prescribing (53%) and monitoring (follow‐up and assessment of medication effects) (36%) – and are frequently linked to weak medication systems and human factors, such as fatigue, poor environmental conditions and staffing shortages (WHO 2017, 2024). The prevalence of preventable medication‐related harm is 7% in LMICs and 4% in high‐income countries, with the highest rates reported in the African and South‐East Asian regions (WHO 2024).

Medication safety, defined as the prevention of medication‐related harm through safe medication practices and systems, requires a comprehensive approach that extends beyond the identification and reduction of medication errors (WHO 2017). Effective nursing leadership and a supportive work environment have emerged as critical components of medication safety (Kowalski et al. 2020). Kowalski et al. (2020) identified five conditions necessary for safe nursing practice: leadership, autonomy, respect and teamwork, adequate resources and organisational support. These factors contribute to a culture that promotes high‐quality care and reduces the likelihood of medication errors. Therefore, nursing leadership plays a vital role in error prevention, managing adverse drug events and supporting error reporting. Existing research on nursing leadership styles has focused primarily on patient outcomes (Ferreira et al. 2022; Sfantou et al. 2017; Wang and Dewing 2021). There is a need to collate and synthesise the evidence around nursing leadership styles and their impact on medication errors in health care settings.

## 2 Background

The United States National Coordinating Council for Medication Error Reporting and Prevention (NCCMERP) defines a medication error as ‘any preventable event that may cause or lead to inappropriate medication use or patient harm while the medication is controlled by the health care professional, patient, or consumer. Such events may be related to professional practice, health care products, procedures, and systems, including prescribing, order communication, product labelling, packaging, nomenclature, compounding, dispensing, distribution, administration, education, monitoring, and use’ (NCCMERP 2025). Considering the wide definition of medication error, not surprisingly, there are several causal factors. The WHO (2017) has clustered these factors into three main categories: (1) factors associated with health care professionals (lack of training, inadequate knowledge and experience, low perception of risk, overworking and fatigue, poor communication between health care team members and patients); (2) factors associated with patients’ characteristics; (3) factors associated with the work environment (issues with the physical work conditions, insufficient resources, unstandardised protocols and procedures, time pressures and distractions/interruptions).

Medication‐related harm originates most frequently during the prescribing and monitoring stages, with prescribing errors accounting for over half of preventable medication‐related harm globally and nearly 80% in LMICs (WHO 2024). Kowalski et al. (2020) emphasised that improving leadership, empowering nurses in decision‐making, ensuring teamwork and respect and addressing resource needs are vital components of a supportive work environment that fosters effective nursing practice. This broader perspective suggests that addressing staffing alone is insufficient; effective leadership and organisational commitment are also necessary to reduce medication errors. This aligns with the WHO Global Patient Safety Challenge: Medication Without Harm, which aims to reduce avoidable medication‐related harm by 50% globally through leadership and system‐wide improvements (WHO 2017).

To understand the role of leadership more clearly, it is important to consider the various leadership styles and behaviours that exist. The foremost leadership styles in the health care/nursing context are defined as transformational leadership: stimulating and assisting the intellectual and creative development of the followers; authentic leadership: transparent and ethical leader behaviour that encourages openness in relationships with followers; ethical leadership: valuing morality and transparency; transactional leadership: guiding and motivating operationalisation of tasks; and servant leadership: concerning to serve followers genuinely (Hult et al. 2023; Singh et al. 2024; Wu et al. 2024). Situational leadership requires leaders to adapt their style based on followers’ development and task demands (Wang et al. 2024) (see Table 1 for additional information on these well‐established styles). Lean leadership, originating from the Toyota production system, focuses on waste reduction and efficiency by leading teams to improve processes stepwise, involve frontline workers and set clear goals (Aij and Teunissen 2017). In addition to these, the negative or ‘dark side’ of leadership has also gained attention. As an emerging concept, toxic leadership involves leaders engaging in systematic and persistent destructive behaviour that can potentially harm employees and organisations. This style is characterised by behaviours such as narcissism, humiliation and abusive supervision, which undermine staff well‐being, reduce trust and collaboration and negatively affect team and organisational performance (Labrague 2021).

**TABLE 1:** Well‐Established Leadership Styles and Definitions (Barkhordari‐Sharifabad and Mirjalili 2020; Singh et al. 2024).

Styles	Definition
Transformational:	Motivates and inspires followers to achieve desired outcomes by focusing on a shared vision and core values. Best for driving change and nurturing innovation.
Transactional:	Focuses on the exchanges between leader and followers, emphasising rewards and punishments based on performance. Suitable for routine tasks and performance.
Servant:	Prioritises the needs of team members and fosters their development, enabling high performance. Embodies humility, empathy and a commitment to serving. Ideal for care environments that prioritise collaboration and team development.
Authentic:	Emphasises building honest relationships with followers. Authentic leaders are self‐aware and genuine. Effective in establishing trust and loyalty within the teams.
Situational:	Leaders must adjust their style to fit and adapt to the development level of followers. The approach allows tailoring strategies to the tasks and the readiness of followers. Beneficial in working with teams possessing varied skills and experience.
Ethical:	Exemplifies integrity, fairness and principled conduct while guiding followers towards responsible behaviour. Encouraging accountability, fostering teamwork and promoting adherence to moral standards.

Primary research in nursing has explored the connection between leadership styles and medication safety, primarily through individual studies that show mixed results, indicating the need for further investigation (e.g. Al‐Rjoub et al. 2024; Kim et al. 2020; Lappalainen et al. 2020). To our knowledge, there is no comprehensive review focusing specifically on nursing leadership styles and medication safety, including medication errors. Three reviews (Wang and Dewing 2021; Ferreira et al. 2022; Sfantou et al. 2017) have addressed related topics as follows: Wang and Dewing (2021) focused on factors such as empowerment, leader–nurse relationships and the quality of the care environment. Ferreira et al. (2022) considered three broad domains: patient outcomes (e.g. patient safety), staff outcomes (e.g. burnout) and institutional outcomes (e.g. turnover and absenteeism). Sfantou et al. (2017) reported on quality‐of‐care measures, including coordinated care, patient satisfaction and adverse events. However, these reviews covered only a limited range of leadership frameworks and were primarily concerned with broader patient safety measures rather than medication safety. Moreover, previous systematic reviews have predominantly focused on quantitative evidence linking leadership styles and patient outcomes. Since these reviews have not explicitly examined leadership styles regarding medication errors, gathering, assessing and synthesising qualitative and quantitative evidence is necessary to better understand nursing leadership's impact on medication errors in health care settings.

### 2.1 Aim

This review aimed to collate, appraise and synthesise evidence on the impact of nursing leadership styles on medication errors.

### 2.2 Research Questions

‐ Which nursing leadership styles have been shown to influence medication errors in health care settings?
‐ How do nursing leadership styles positively or negatively influence medication errors in health care settings?
‐ Which mediating factors along nursing leadership styles may influence medication errors in health care settings?

## 3 Methods

### 3.1 Design

This review followed the Joanna Briggs Institute (JBI) methodology for convergent segregated mixed‐methods systematic reviews (Lizarondo et al. 2024; Stern et al. 2020). A mixed‐methods design was selected in order to answer the research question and address the heterogeneous nature of this body of evidence. Mixed‐methods systematic reviews reflect the complexity of research questions in health care and assist with the depth and breadth of understanding (Stern et al. 2020). The review was reported following the Preferred Reporting Items for Systematic Reviews and Meta‐Analyses (PRISMA) guidelines (Page et al. 2021) to enhance transparency and replicability. This systematic review's protocol was registered in PROSPERO (registration number CRD42023380426).

### 3.2 Search Methods

To identify relevant studies, a systematic literature search strategy was employed. Article retrieval was performed via databases including MEDLINE, CINAHL, Scopus, PubMed, Web of Science, EMBASE, PsycINFO and the search engine Google Scholar between January 2014 and April 2025. The time frame of 2014–2025 was selected to capture contemporary evidence reflecting recent developments in nursing leadership and medication safety. The search terms used were “nurse” OR “nursing” AND “leadership” AND “medication error” (detailed search strings are provided in Supplementary File S1). Two reviewers independently screened titles and abstracts, followed by full‐text screening to assess eligibility, using Covidence software for management and duplicate removal. Any disagreements were resolved through discussion among reviewers to ensure consensus.

### 3.3 Inclusion/Exclusion Criteria

The review objectives guided the inclusion and exclusion criteria and were structured to ensure methodological transparency and replicability. Studies were selected based on the following eligibility criteria using the Population, Intervention, Comparison, Outcomes and Study design (PICOS) framework (Lizarondo et al. 2024). Eligible studies included those involving registered nurses in health care settings and examining nursing leadership styles as the primary focus. Studies involving patients within these settings were also included. Studies focusing on non‐registered or non‐qualified nurses – student nurses, nursing assistants, practical nurses or vocational nurses – or those involving multi‐professional samples without nurse‐specific findings were excluded. No specific comparator or control was required for inclusion. Studies were considered if conducted in primary, secondary, or tertiary care settings, including acute care, paediatric and psychiatric contexts. Only empirical studies in English using qualitative, quantitative or mixed‐methods designs were included. Literature reviews, dissertations, abstracts and books were excluded.

### 3.4 Quality Appraisal

Two reviewers independently evaluated eligible studies for methodological quality using established critical appraisal tools selected according to the study design. The JBI Critical Appraisal Checklists were applied to cross‐sectional and quasi‐experimental studies. The Mixed Methods Appraisal Tool (MMAT) was employed for mixed‐methods studies (Hong et al. 2018), and the Critical Appraisal Skills Programme (CASP) checklist was utilised for cohort studies (CASP 2024). Quality improvement projects were assessed using the Quality Improvement Minimum Quality Criteria Set (QI‐MQCS), a tool tailored for QI interventions that cover 16 content domains (Hempel et al. 2015).

To ensure consistency and rigour in methodological assessment, each reviewer independently completed the appropriate checklist and documented their evaluation of each study's quality. The reviewers then discussed their appraisals in joint review meetings to reach a consensus on methodological strengths, limitations and potential bias. Quality appraisals were conducted to describe the quality of the studies rather than to exclude low‐quality studies or selectively include high‐quality ones (Lizarondo et al. 2024; Stern et al. 2020).

### 3.5 Data Extraction and Synthesis

After finalising the list of eligible studies, two reviewers extracted data independently using a structured extraction table developed for this review. Extracted data included author(s), year, country, aim, sample characteristics, study design, data collection and analysis methods, and key findings. The JBI convergent segregated approach (Stern et al. 2020) was used to synthesise quantitative and qualitative data separately before integrating results during interpretation. Quantitative data were synthesised narratively using Popay et al.’s (2006) narrative synthesis framework, involving four key stages: developing a theoretical understanding of how leadership styles influence medication safety; conducting a preliminary synthesis to identify patterns and associations; exploring relationships to explain variations across studies and assessing the robustness of the synthesis. Qualitative data were summarised descriptively, and where appropriate, quantitative results were transformed into qualitative descriptions to facilitate integration with qualitative findings, providing contextual and explanatory insights to enrich interpretation.

## 4 Results

### 4.1 Search Results and Study Selection

**FIGURE 1:**
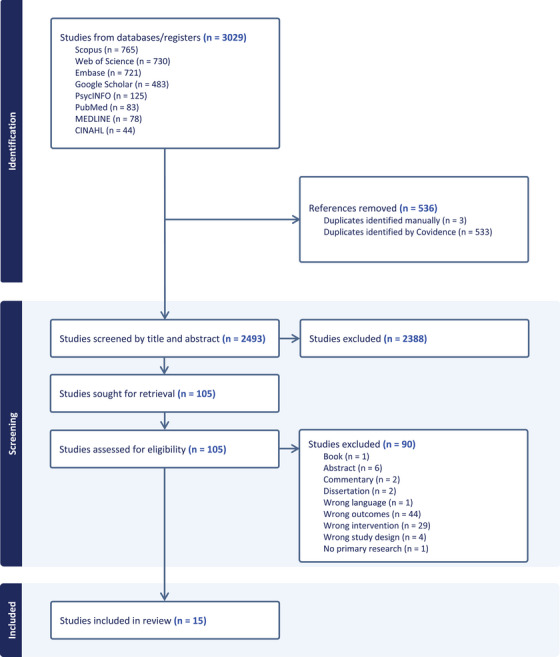
PRISMA flow diagram.

The literature search yielded 3,029 references. Of these, 536 were removed as duplicates, and 2,388 were excluded during title and abstract screening. The remaining 105 papers were read in full, and 90 were excluded, resulting in the final inclusion of 15 studies. The screening process according to PRISMA is shown in Figure 1.

### 4.2 Quality Appraisal Findings

The cross‐sectional studies clearly described sampling methods ranging from random to convenience sampling. These studies adequately reported on sample recruitment, data collection instruments and the validity of measurement tools and statistical analyses. Confounding variables were identified in only one study (Labrague 2021). The retrospective cohort study by Al‐Rjoub et al. (2024) and quality improvement studies (Bryant and Yoder 2021; Smith‐Love 2022) provided sufficient detail on sample selection, outcome measurement and analytic methods. The quasi‐experimental study (Mohiuddin and Mohteshamuddin 2020) clearly described intervention implementation, participant allocation and follow‐up procedures. Based on these assessments, all included studies were considered methodologically sound and appropriate for inclusion in the data synthesis and narrative review (outcome of appraisal of included studies in Supplementary File S2).

### 4.3 Study Characteristics, Methods and Approaches Used for Examining the Relationships

The included studies, published between 2015 and 2024, were conducted in nine countries: the United States (*n* = 5), Jordan (*n* = 2) and one study each from Iran, Saudi Arabia, the United Kingdom, South Korea, Finland, the Philippines and the United Arab Emirates. Settings included a range of hospitals – acute care, community, teaching and a Magnet hospital – with general wards, medical–surgical wards, cardiac units, and critical care units and hospital‐affiliated settings as ambulatory surgical and specialised centres.

Most studies (*n* = 10) employed cross‐sectional survey designs to examine the relationships between leadership styles and medication safety outcomes. Commonly used instruments included the Multifactor Leadership Questionnaire (*n* = 9), the Hospital Survey on Patient Safety Culture (*n* = 2) and various tools for assessing medication error reporting and safety climate. Two studies adopted quality improvement approaches involving observational data and performance metrics (Bryant and Yoder 2021; Smith‐Love 2022); one study used a quasi‐experimental design to evaluate the implementation of a leadership model combining Kotter's change theory and situational leadership (Mohiuddin and Mohteshamuddin 2020); one study employed a retrospective cohort design including a cross‐sectional survey (Al‐Rjoub et al. 2024); and one mixed‐methods study evaluated a continuing professional education programme on safe medication administration, using document analyses and observational data to examine leadership styles and incident reporting (Browne et al. 2021).

Sampling strategies varied across the included papers. Convenience sampling was most frequent, while others employed random or stratified random sampling to enhance generalisability. Sample sizes ranged from small groups (e.g. 40–60 participants) to 1,053 nurses (Labrague 2021). Across all 15 studies, a total of 3,680 nurse participants were involved. Data collection methods included structured surveys (*n* = 15), observations (*n* = 3) document or chart reviews (*n* = 2) and medication incident reports (*n* = 4). Several studies combined multiple data sources to triangulate leadership effects on medication safety outcomes (Table 2).

**TABLE 2:** Characteristics and summary of included studies (*n* = 15).

Author, year, country	Aim	Design	Setting, participants, sampling	Data collection, tools, analyses	Leadership styles	Medication safety aspect	Key finding
Al‐Oweidat et al. (2023), Jordan	To explore nurses’ perceptions of the influence of nurse managers’ leadership behaviours and organisational culture on patient safety incident reporting practices.	Cross‐sectional	15 hospitals 325 nurses Convenience sampling	Survey (Leadership Practices Inventory, Professional Organisational Culture Scale, Incident Reporting Questionnaire) Correlation analysis	Transformational	Medication errors reporting	A significant positive relationship between transformational leadership and organisational culture, and incident‐reporting practices, including medication errors. Additionally, organisational culture and incident‐reporting practices.
Al‐Rjoub et al. (2024), Jordan	To examine the effect of leadership style at different managerial levels on nursing care performance and patient outcomes.	Retrospective cohort including cross‐sectional survey	Public educational hospital (the general ward and the critical care unit) 60 nurses and 300 patients Nurses: Stratified random sampling Patients: Not reported	Survey, observing practice and reviewing health records (Multifactor Leadership Questionnaire, Nursing performance indicators, Standardised chart review form) Regression analysis	Transformational Transactional	Medication rights Medication errors	Nurses with transactional leaders were more likely to follow standardised care protocols, including medication rights. Multivariate analyses showed that medication errors did not show a significant relationship with leadership style.
Barkhordari‐Sharifabad et al. (2020), Iran	To determine the level of ethical leadership from the nurses’ perspective and its effect on nursing error and error reporting.	Cross‐sectional	Teaching hospitals’ medical–surgical wards 171 nurses Random sampling	Survey (Ethical leadership questionnaire, Nursing errors questionnaire, Error reporting questionnaire) Correlation analysis	Ethical	Medication errors Error reporting	A small but statistically significant negative correlation between ethical leadership and medication errors, and a significant positive correlation with error reporting.
Bryant and Yoder (2021) USA	To determine the impact of the Lean Management System on unfinished nursing care	Quality improvement	Two medical–surgical units in an academic health system Not reported	Review of unfinished nursing care (Nursing Quality Indicators, Missed Care Survey) Descriptive analysis	Lean	Administering medications on time	Following the implementation of the Lean Management Model in both units, the prevalence of unfinished nursing care, including medication errors, decreased, declining from 49% to 25% in one unit and from 26% to 17% in the other.
Browne et al. (2021), UK	To explore a continuing professional education programme on the safe administration of medication and how new knowledge and skills are transferred into clinical practice.	Mixed‐method	Acute care hospital 152 nurses Not reported	Document analyses, observations (Qualitative: documents; Quantitative: observation tool, reported critical incidents and nursing care metrics) Thematic analysis	Transformational Laissez‐Faire Autocratic	Medication incident reports	The ward with the transformational leader had the highest medication incidents (50), linked to a culture of openness in reporting errors. The laissez‐faire ward reported 20 errors, and the autocratic ward reported 15.
Farag and Anthony (2015), USA	To examine the relationship between work environment characteristics (leadership style and safety climate) and nurses’ willingness to report medication errors.	Cross‐sectional	Four ambulatory surgical settings 40 registered nurses Not reported	Survey (Multifactorial Leadership Questionnaire, Patient Safety Climate in Health care Organisations Tool) Regression analysis	Transformational Transactional Passive avoidant	Willingness to report medication errors	Three leadership styles explained 44% of the variance in nurses’ willingness to report medication errors. Passive avoidant had the only significant negative effect.
Farag et al. (2017a), USA, Canada	To examine the relationship among work environment (nurse manager leadership style and safety climate), social capital (warmth and belonging relationships and organisational trust) and nurses’ willingness to report medication errors.	Cross‐sectional	Three community and two critical access hospitals 71 nurses Convenience sampling	Survey (Safety Climate Survey, Multifactorial Leadership Questionnaire, Organisational Climate Survey) Correlation analysis	Transformational Transactional	Willingness to report medication errors	Willingness to report errors was weakly positively associated with transactional nurse management leadership style but not with transformational leadership style. Neither warmth and belonging relationships nor organisational trust was related to willingness to report at a statistically significant level.
Farag et al. (2017b), USA	To examine the effect of leadership style, unit climate and safety climate on nurses’ safe medication practices.	Cross‐sectional	Magnet hospital (5 critical care and 11 medical–surgical units) 246 registered nurses Convenience sampling	Survey (Multifactorial Leadership Questionnaire, Organisational Climate Questionnaire, Agency for Health care Research and Quality Hospital Survey, Institute of Safe Medication Practice) Path analysis	Transformational Transactional Passive avoidant	Willingness to report medication errors Safe medication administration	Three leadership styles indirectly (through the safety climate dimension of nonpunitive response to errors) explained 2% of the variance in nurses’ willingness to report medication errors. Transactional and passive‐avoidant leadership styles indirectly (through organisational learning) explained 6% of variance in safe medication administration.
Farag et al. (2019), USA	To examine the effect of organisational (nurse manager's leadership style and safety climate) and social (warmth and belonging climate and organisational trust) factors on nurses’ safety motivation (willingness to report medication errors).	Cross‐sectional	Acute and critical care units 144 registered nurses Systematic random sampling (every 15th)	Survey (Safety Culture Survey, Multifactorial Leadership Questionnaire, Organisational Climate Questionnaire, Organisational Trust Instrument) Path analysis	Transformational Transactional	Medication errors reporting	Transformational leadership style significantly negatively affects nurses’ safety motivation, meaning they are less motivated to report medication errors. However, it also demonstrates a positive indirect effect through improvements in the safety climate – particularly error feedback and nonpunitive response to error – resulting in a nonsignificant total effect.
Hamdan et al. (2024), Saudi Arabia	To investigate the mediating effect of patient safety culture between the relationship of transformational leadership and safety practices among nurses.	Cross‐sectional	Three hospitals and two specialised centres 200 nurses Random sampling	Survey (Multifactor Leadership Questionnaire, Hospital Survey on Patient Safety Culture, Nursing Safety Practices Scale) Structural equation modelling analysis	Transformational	Medication administration safety	Transformational leadership has a strong total effect on nursing safety practices, including medication administration safety, with both a positive and significant direct effect and an indirect effect through patient safety culture.
Kim et al. (2020), Republic of Korea	To investigate the mediating role of the perceived benefits of using a medication safety system in the relationship between transformational leadership and the medication‐error management climate.	Cross‐sectional	11 hospitals 158 registered nurses Non‐probability sampling method	Survey (Multifactorial Leadership Questionnaire, Korean version of the Error Management Climate Questionnaire) Regression analysis	Transformational	Medication error management climate	Transformational leadership has a significant effect on the medication error management climate, both directly and indirectly through the perceived benefits of using medication safety systems (such as electronic reporting and barcode systems).
Labrague (2021), Philippines	To assess the impact of toxic leadership behaviours among nurse managers on nurse‐reported adverse events and quality of care.	Cross‐sectional	20 hospitals 1053 registered nurses Not reported	Survey (Toxic Leadership Behaviours of Nurse Managers Scale, The Adverse Patient Events Scale, The single‐item quality‐of‐care measure) Regression analysis	Toxic	Medication errors	Toxic leadership behaviours were strongly associated with increased medication errors.
Lappalainen et al. (2020), Finland	To describe medication safety, transformational leadership and their relationship.	Cross‐sectional	Three hospitals 161 registered nurses Not reported	Survey (Transformational Leadership Scale, Medication Safety Scale) Regression analysis	Transformational	Medication safety	Transformational leadership moderately statistically significantly correlates with medication safety.
Mohiuddin and Mohteshamuddin (2020), United Arab Emirates	To assess whether implementing a combination of Kotter's change model and Hersey and Blanchard's situational leadership model will improve the self‐reporting of medication errors.	Quasi‐experimental	Not reported 21 physicians, 19 nurses Not reported	Monitoring medication error reports (Medication error reports) Descriptive analysis	Situational	Medication errors	The situational leadership model improved self‐reporting of medication errors. The first month had zero reports; the second had three (one near miss, two self‐reported) and the third had two self‐reported cases.
Smith‐Love (2022), USA	To reduce near‐miss medication events, address system failures and elevate individual performance through evidence‐based strategies that support accountability, open communication and stakeholder engagement.	Quality improvement	Cardiac unit (30 beds) 49 nurses Not reported	Medication management electronic scanning practices: medication administration and patient identification (Medication management electronic system) Descriptive analysis	Transformational	Medication errors	Transformational leadership guided and engaged staff in reducing medication errors with sustainability over 2 years, with scanning improving from 96.4% to 98.1% in one month, and surpassing 99% within 3 months. These improvements were sustained above the 97% benchmark for over 2 years.

### 4.4 Leadership Styles Assessed in the Included Studies

A wide range of leadership styles was assessed across the studies. Transformational leadership was most commonly examined, appearing in 11 studies (Al‐Oweidat et al. 2023; Al‐Rjoub et al. 2024; Farag and Anthony 2015; Farag et al. 2017a, 2017b, 2019; Hamdan et al. 2024; Kim et al. 2020; Lappalainen et al. 2020; Browne et al. 2021; Smith‐Love 2022). Transactional leadership was assessed in five studies (Farag and Anthony 2015; Farag et al. 2017a, 2017b, 2019; Al‐Rjoub et al. 2024) and passive‐avoidant leadership was assessed in two studies (Farag et al. 2017b; Al‐Rjoub et al. 2024). Other leadership styles explored included ethical leadership (Barkhordari‐Sharifabad et al. 2020), situational leadership (Mohiuddin and Mohteshamuddin 2020), autocratic and laissez‐faire (Browne et al. 2021), Lean leadership (Bryant and Yoder 2021) and toxic leadership (Labrague 2021).

### 4.5 The Focus and Impact of Leadership Styles on Medication Safety

Leadership styles appeared to directly and indirectly influence medication safety outcomes, including error reporting, medication rights adherence, administration practices and safety climate. Transformational leadership showed positive associations with safe medication practices. For example, it significantly improved incident‐reporting practices, including medication errors (*r* = .131, *p*  < .001) (Al‐Oweidat et al. 2023); supported a favourable medication error management climate both directly (*β* = .55, *p* < .001) and indirectly through perceived system benefits (*β* = .53, *p* < .001) (Kim et al. 2020); and guided sustained reductions in medication errors, with electronic scanning compliance in one study showing an increase from 96.4% to over 99%, and maintaining above 97% for two years (Smith‐Love 2022). Similarly, transformational leadership was linked to improved medication safety practices in both direct (*β* = .216, *p* < .001) and mediated (*β* = .420, *p* < .001) pathways (Hamdan et al. 2024), as well as a moderately strong correlation with medication safety (*r* = .541, *p*  < .001) (Lappalainen et al. 2020). However, one study found a negative impact on safety motivation, with transformational leadership reducing willingness to report medication errors (*β* = –.30, *p* < .001), though a positive indirect effect offset this via improvements in safety and social climate (*β* = .27, *p* < .001) (Farag et al. 2019).

Transactional leadership had a mixed impact. It was associated with adherence to medication rights, as nurses under transactional leaders were more likely to follow standardised care protocols (*χ^2^* = 25.2, *p* < .001) but had no significant direct effect on medication errors (*OR* = 0.3, *p* = .05) (Al‐Rjoub et al. 2024). It was also associated with increased willingness for error reporting (*r* = .28, *p* = .01) (Farag et al. 2017a). Both transactional and passive‐avoidant leadership styles were found to indirectly explain 6% of the variance in safe medication administration (Farag et al. 2017b). Passive‐avoidant leadership additionally showed adverse effects, with a significant negative relationship with nurses’ willingness to report medication errors (*β* = –.45, *p* = .01) (Farag and Anthony 2015).

Ethical leadership was linked to reduced medication errors (*r* = –.275, *p* < .001) and increased reporting (*r* = .206, *p* = .007) (Barkhordari‐Sharifabad et al. 2020). As applied through Kotter's model, situational leadership improved self‐reporting behaviour on medication errors over time, increasing from zero to three reports in the second month and two in the third month of the study (Mohiuddin and Mohteshamuddin 2020). Toxic leadership showed a strong association with medication errors (*β* = .708, *p* < .001), highlighting the detrimental impact of poor leadership on safety (Labrague 2021). Lean leadership approaches reduced unfinished care, including timely medication administration, with errors declining from 49% to 25% and 26% to 17% in two separate units (Bryant and Yoder 2021). In a comparative setting, transformational leadership was associated with the highest reported incidents (*n* = 50), attributed to a more open culture of error reporting (Browne et al. 2021). At the same time, laissez‐faire and autocratic wards had 20 and 15 reported incidents, respectively (Browne et al. 2021).

### 4.6 Mediating Factors in the Work Environment

Seven studies explored mediating mechanisms between leadership styles and medication safety. Safety culture or safety climate was the most frequently examined mediator, appearing in five studies. According to Hamdan et al. (2024), transformational leadership directly and indirectly affects medication safety practices, mediated by patient safety culture. Similarly, Farag et al. (2017b) demonstrated that the safety climate dimension of nonpunitive response to error mediated the impact of transformational, transactional and passive‐avoidant leadership on nurses’ willingness to report medication errors. Organisational learning – in which work processes are regularly reviewed, changes are made to prevent mistakes and those changes are evaluated – emerged as another key mediator, particularly in Farag et al. (2017b), which explained the indirect impact of transactional and passive‐avoidant leadership styles on safe medication administration. Organisational culture was a mediating factor in Al‐Oweidat et al. (2023), where transformational leadership positively influenced incident‐reporting practices. Social capital factors, including warmth, belonging and organisational trust, were examined in two studies. Farag et al. (2019) found that warmth and belonging significantly indirectly affected safety motivation. In contrast, Farag et al. (2017a) found no significant associations, suggesting an inconsistent effect of social factors across settings. Finally, perceived system benefits, such as the utility of medication safety systems (e.g. barcode scanners or reporting tools), mediated the relationship between transformational leadership and a favourable medication error management climate in Kim et al. (2020), highlighting the role of technological adoption in reinforcing safety practices. These mediators demonstrate how leadership shapes individual behaviour and the broader environmental and organisational conditions that support medication safety.

## 5 Discussion

This review has shown that nursing leadership can influence medication safety through complex, interrelated pathways. Different leadership styles had distinct and often divergent effects on medication error reporting, adherence to medication rights and administration practices. These effects were also shaped by underlying mechanisms such as patient safety culture, organisational learning, social capital and system usability. The findings from this review highlight the need for more integrated leadership approaches that foster safer nursing care.

### 5.1 Leadership Approaches Shaping Medication Safety

Transformational leadership emerged from the findings as a generally positive driver of improvements in error reporting, safety climate and the reduction of medication errors. Consistent with this, Wu et al. (2024) identified transformational leadership as the most widely studied leadership style in health care and reported strong links with improved patient safety culture and quality of care. However, our review also indicates that transformational leadership can have mixed effects on medication safety motivation. Specifically, some evidence suggests reduced willingness among nurses to report medication errors. Barrachina and González‐Chordá (2020) argue that the emphasis of transformational leadership on nonpunitive and rewarding actions fosters greater nurse participation. Nonetheless, passive (hesitant to report) and reactive (conditional/selective to report) nurses may initially underreport errors under a transformational leadership style. They suggest an evolutionary process where nurses’ reporting behaviour matures from passive to active (proactive to report), reflecting growth towards a positive safety culture. This behavioural shift supports the theory of planned behaviour, where intention and perceived control evolve with organisational support (Ajzen 1991). The higher incidence of reported errors under transformational leadership, compared to more autocratic or laissez‐faire environments, likely reflects a culture of transparency, rather than poorer safety (Browne et al. 2021).

Transactional leadership exhibited mixed outcomes, associating positively with protocol adherence and error reporting, but not consistently predicting medication errors. Hult et al. (2023) similarly noted that task‐oriented (transactional) leadership style is linked to both positive and negative outcomes (such as less/more turnover, better/lower performance) for the organisation and health care professionals and positive outcomes (such as fewer adverse events, shorter length of stay) for patients. This highlights how the impact of transactional leadership varies depending on the context and nurse behaviour. Richards (2020) also highlighted the strengths and limitations of transactional leadership, acknowledging its utility in achieving short‐term goals, while recommending a combination with other leadership styles to maximise effectiveness. In alignment with this perspective, Nurmeksela et al. (2021) demonstrated that nurse managers’ everyday organisational practices – how they coordinate and support staff – shape nurses’ job satisfaction, patient satisfaction and medication error. By influencing the safety and motivation of the work environment, these managerial factors help explain why transactional leadership, centred on task management and structure, produces variable outcomes depending on workload allocation and perceived support. These findings, collectively, support combining transactional leadership's task orientation with transformational leadership's focus on motivation and organisational culture to optimise outcomes.

Few studies have examined the impact of ethical and situational leadership styles on medication safety. Ethical and situational leadership approaches benefit medication safety by reducing errors and encouraging self‐reporting behaviours, as revealed by the studies included in this review (Barkhordari‐Sharifabad et al. 2020; Mohiuddin and Mohteshamuddin 2020). Moraca et al. (2025) also demonstrated that, in clinical practice, ethical and authentic leadership styles mediate the relationship between organisational constraints and errors, highlighting how head nurses who adopt these leadership styles can effectively mitigate organisational barriers to enhance patient safety. Additionally, as indicated by Wang et al. (2024), situational leadership theory emphasises adapting leadership styles to followers’ needs and maturity to support nurse leadership development, facilitate organisational goal achievement and enhance follower satisfaction.

Aside from well‐known and well‐established styles, our review has revealed other critical emerging leadership approaches. As a quality improvement strategy, Lean leadership positively influences timely medication administration (Bryant and Yoder 2021). Complementing these findings, Mendes and França (2025) demonstrate that Lean thinking enhances health care efficiency, reduces costs and improves patient safety by focusing on risk management and process optimisation. By contrast, a significant contemporary concern is the ‘dark side’ of leadership, with toxic leadership style having adverse effects, including an increase in medication errors (Labrague 2021). These harmful impacts align with broader literature linking destructive leadership to burnout, psychological distress and environments prone to errors (Wu et al. 2024). Hult et al. (2023) corroborate these findings by identifying destructive leadership styles as strongly associated with adverse organisational and staff outcomes, calling attention to the need to avoid such leadership styles as they have detrimental effects on medication safety and health care quality.

### 5.2 Mechanisms Linking Leadership to Medication Safety

Understanding the mechanisms behind the leadership effects presented earlier is critical. Seven studies identify mediating pathways such as patient safety culture, organisational culture and safety climate dimensions, including a nonpunitive response to errors, organisational learning, technological adoption and perceived system benefits. These mechanisms converge with broader organisational behaviour theories, which suggest that leadership operates through direct supervision and by shaping the social and cultural context in which nurses make safety decisions (Huang et al. 2024). Huang et al. (2024) further clarify these pathways, demonstrating that management leadership influences safety culture by mediating teamwork climate, stress recognition and working conditions. This suggests that leadership shapes staff attitudes and fundamentally structures the environment where safety behaviours emerge. Consistent with this, Seljemo et al. (2020) found, in a Norwegian nursing context, that transformational leadership explained nearly half of the variance in patient safety culture and significantly influenced staff perceptions of safety. While this underscores the essential role of leadership in shaping positive safety environments, it does not take into account the contribution of other organisational determinants. Social capital factors – warmth, belonging and trust – show inconsistent mediation effects, reflecting contextual variability in influencing safety motivation and error reporting (Farag et al. 2017a, 2019). Hult et al. (2023) identified trust in the manager as a mediating pathway in translating leadership styles into improved outcomes, indicating that other mediators, such as structural empowerment and job satisfaction, also play a role. This suggests that these mediators require further exploration to clarify their role.

### 5.3 Towards Integrating Leadership Styles for Safer Nursing Care Environments

Our review has revealed that nursing leadership directly influences medication safety through practice and error reporting and is indirectly mediated by workplace culture, climate, learning and social factors. Insights from Barrachina and González‐Chordá (2020) elucidate the nuanced roles of leadership styles on nurse error reporting behaviour, supporting the view that a combination of transformational and active transactional leadership elements may best enhance medication safety. Supporting this, van Elp et al. (2022) demonstrated that Lean leadership, which blends transformational and transactional behaviours, fosters continuous improvement capabilities in health care teams, underscoring the necessity for hybrid leadership to achieve sustainable quality and safety outcomes. Richards (2020) also advocates combining leadership styles to optimise effectiveness, aligning with these conclusions. Kowalski et al. (2020) further suggest that authentic leadership, integrated with transformational leadership, provides the necessary support and resources to nurses, exemplifying the value of combined leadership approaches. This indicates that authentic leadership is emerging as another promising style that, independently or combined with transformational leadership, may foster nurses’ safety actions, reduce adverse patient events and enhance care quality. Wu et al. (2024) emphasised the rise of authentic leadership in recent years as a response to workplace moral demands, linking it to increased psychological safety, open communication and the development of social capital among nursing teams. Wu et al. (2024) also assert that future leadership research should prioritise core ethical, authentic and empowering themes while addressing emerging concerns like toxic leadership to support resilient, psychologically safe and high‐performing health care environments. These perspectives align with Anderson and Sun's (2017) call for an integrated ‘full‐range’ leadership model that captures the overlapping nuances of leadership styles. To strengthen future leadership interventions, studies should adopt theory‐driven, mixed‐method designs that more effectively capture contextual variation and long‐term cultural shifts. Further research integrating qualitative and quantitative evidence is necessary to deepen understanding and develop tailored interventions across diverse health care settings.

### 5.4 Strengths and Limitations

This review comprehensively analysed how different nursing leadership styles impact medication safety, synthesising evidence from multiple rigorous studies. Its strength lies in integrating consistent and contradictory findings to reveal the complex mechanisms – such as safety culture and organisational learning – mediating leadership's effects on safety outcomes. However, limitations include a predominant focus on hospital‐based research, which may limit the generalisability to other care settings, like community or private health care. The relative scarcity of studies on emerging leadership approaches, such as situational and Lean leadership, restricts the ability to assess their effectiveness fully. Additionally, variations in study designs, populations and measurement tools create challenges for direct comparisons and warrant cautious interpretation of some findings. Finally, the review was limited to studies published in English, which may have excluded relevant findings in other languages.

## 6 Conclusion

Nursing leadership plays a critical and multifaceted role in promoting medication safety. Transformational leadership shows positive effects by improving error reporting, safety climate and staff engagement. However, its influence on safety motivation can be complex and context‐dependent. Transactional leadership contributes to better adherence to medication protocols but has an inconsistent impact on reducing medication errors. Importantly, toxic and passive‐avoidant leadership styles are strongly linked to increased errors and diminished reporting, underscoring the need to avoid these detrimental approaches. Emerging leadership styles like Lean leadership and authentic style appear promising in enhancing medication safety but require further empirical validation. Overall, a hybrid leadership model that combines transformational, transactional and Lean elements appears most effective in fostering a culture of safety, open communication and continuous learning within nursing teams. To further advance understanding, future research could adopt study designs that include randomised controlled trials and mixed‐methods approaches, involving promising approaches such as Lean and authentic. Future studies could also be extended to regions such as Australia, Africa and South America and explore settings beyond hospitals, including community centres.

## 7 Implications for Nursing and Health Policy

Nursing leadership must evolve towards integrated and adaptive approaches to reduce medication errors and improve patient outcomes. Leaders should prioritise transformational and authentic leadership behaviours, encouraging psychological safety, transparency and shared accountability. Incorporating transactional leadership's task orientation can enhance adherence to safety protocols when combined with relational leadership elements. Nurse managers are encouraged to foster organisational learning, promote open communication and use data‐driven tools like safety dashboards for ongoing monitoring and improvement. Lean leadership principles can further streamline processes and reduce unfinished care. Caution is necessary to identify and mitigate toxic leadership behaviours, significantly harming safety culture and staff well‐being. At this point, it is essential to recognise that followers can play an active role in neutralising the effects of toxic leaders through practical workarounds – such as building alternative support networks and finding creative ways to navigate challenges – and continuous learning, rather than remaining passive recipients of harmful behaviours (Milosevic et al. 2020). Organisational strategies – including policies that outline expected behaviours in the workplace, open communication and resilience‐building interventions – could also help prevent and mitigate the impact of toxic leadership (Labrague 2021). Finally, investing in leadership development programmes, particularly in promising styles, such as authentic leadership (Dirik and Seren Intepeler 2024) and cultivating situational adaptability and hybrid leadership skills (Singh et al. 2024), will be key to sustaining long‐term medication safety improvements across diverse health care settings. This comprehensive, adaptive approach aligns with Anderson and Sun's (2024) argument for a hierarchical and situationally responsive framework of leadership behaviours, integrating foundational behaviours into broad, practical leadership dimensions.

## Author Contributions

Study design: HFD, NE. Data collection: HFD, NE, CT. Data analysis: HFD, NE, CT. Study supervision: NE. Manuscript writing: HFD, NE. Critical revisions for important intellectual content: HFD, NE, CT.

## Funding

The authors have nothing to report.

## Ethical Considerations

No ethical approval was required.

## Conflicts of Interest

The authors declare no conflicts of interest.

## Supporting information




**Supporting File 1**: inr70166‐sup‐0001‐SupMat‐File‐1.docx

**Supporting File 2**: inr70166‐sup‐0002‐SupMat‐File‐2.xlsx

## Data Availability

The data that support the findings of this study are available from the corresponding author upon reasonable request.
