# Supplementary material for: The Influence of Nursing Leadership Styles on Medication Safety: A Mixed‐Methods Systematic Review
Source: Int Nurs Rev. 2026 Feb 23;73(1):e70166. doi: 10.1111/inr.70166 (PMC12927690; doi:10.1111/inr.70166)
Supplement: Supplementary file 1 — Supporting File 1: inr70166‐sup‐0001‐SupMat‐File‐1.docx [file INR-73-0-s001.docx]

**APA PsycInfo**

1 exp Nurses/ or exp Nursing/ or Nurs*.mp. 130932

2 patient*.mp. 865478

3 hospital*.mp. 216239

4 healthcare.mp. 70679

5 2 or 3 or 4 975308

6 exp Leadership/ or leader*.mp. or exp Leadership Style/ 120939

7 'leadership approach'.mp. 394

8 6 or 7 120939

9 medication*.mp. 106774

10 medication safe*.mp. 376

11 medication error*.mp. 1095

12 drug error*.mp. 31

13 medication administration*.mp. 689

14 medication administration error*.mp. 82

15 order* error*.mp. 200

16 prescribing error*.mp. 74

17 prescription error*.mp. 39

18 transcription error*.mp. 61

19 transcribe error*.mp. 0

20 dispensing error*.mp. 28

21 medication event*.mp. 233

22 medication incident*.mp. 36

23 adverse drug event*.mp. 434

24 medication harm*.mp. 14

25 medication injur*.mp. [mp=title, abstract, heading word, table of contents, key concepts, original title, tests & measures, mesh word] 0

26 near miss*.mp. 620

27 error* report*.mp. 259

28 medication error* report*.mp. 44

29 incident.mp. 17220

30 incident report*.mp. 735

31 event report*.mp. 488

32 9 or 10 or 11 or 12 or 13 or 14 or 15 or 16 or 17 or 18 or 19 or 20 or 21 or 22 or 23 or 24 or 25 or 26 or 27 or 28 or 29 or 30 or 31 124687

33 1 and 5 and 8 and 32 189

34 limit 33 to yr="2014 -Current" 115

**EMBASE**

1 exp Nurses/ or exp Nursing/ or Nurs*.mp. 900925

2 patient*.mp. 13013382

3 hospital*.mp. 3367228

4 healthcare.mp. 629271

5 2 or 3 or 4 14066829

6 exp Leadership/ or leader*.mp. or exp Leadership Style/ 156992

7 'leadership approach'.mp. 129

8 6 or 7 156992

9 medication*.mp. 730714

10 medication safe*.mp. 5621

11 medication error*.mp. 23975

12 drug error*.mp. 792

13 medication administration*.mp. 5789

14 medication administration error*.mp. 582

15 order* error*.mp. 401

16 prescribing error*.mp. 2621

17 prescription error*.mp. 1108

18 transcription error*.mp. 674

19 transcribe error*.mp. 1

20 dispensing error*.mp. 734

21 medication event*.mp. 1054

22 medication incident*.mp. 371

23 adverse drug event*.mp. 8450

24 medication harm*.mp. 116

25 medication injur*.mp. [mp=title, abstract, heading word, drug trade name, original title, device manufacturer, drug manufacturer, device trade name, keyword heading word, floating subheading word, candidate term word] 25

26 near miss*.mp. 4672

27 error* report*.mp. 2336

28 medication error* report*.mp. 900

29 incident.mp. 144563

30 incident report*.mp. 5041

31 event report*.mp. 7734

32 9 or 10 or 11 or 12 or 13 or 14 or 15 or 16 or 17 or 18 or 19 or 20 or 21 or 22 or 23 or 24 or 25 or 26 or 27 or 28 or 29 or 30 or 31 881227

33 1 and 5 and 8 and 32 1073

34 limit 33 to yr="2014 -Current" 721

**MEDLINE**

1 nurse.mp. or Nurses/ 246257

2 nursing.mp. or Nursing/ 690324

3 leadership.mp. or Leadership/ 88351

4 Medication Errors/ 14918

5 1 or 2 780682

6 3 and 4 135

7 5 and 6 63

**Web of Science**

ALL=(((“Nurs*”) AND (“patient*” OR “hospital*” OR “healthcare”) AND (“leader*” OR “leadership*” OR “leadership style*” OR “leadership approach*” OR “leadership type*”) AND (“medication*” OR “medication safe*” OR “medication error*” OR “drug error*” OR “medication administration*” OR “medication administration error*” OR “order* error*” OR “prescri* error*” OR “transcri* error*” OR “dispens* error*” OR “medication event*” OR “medication incident*” OR “adverse drug event*” OR “medication harm*” OR “medication injur*” OR “near-miss*” OR “near miss*” OR “error* report*” OR “medication error* report*” OR “incident” OR “incident report*” OR “event report*”)))

**Scopus**

ALL ( ( nurse or nursing ) and ( leadership ) and ( medication error ) ) AND PUBYEAR > 2013 AND PUBYEAR < 2024 AND PUBYEAR > 2013 AND PUBYEAR < 2024 AND ( LIMIT-TO ( SUBJAREA , "NURS" ) ) AND ( LIMIT-TO ( DOCTYPE , "ar" ) ) AND ( LIMIT-TO ( LANGUAGE , "English" ) )

**Google Scholar, CINAHL, PubMed**

(((nurses) OR (nursing)) AND (leadership)) AND (medication error)
